# Safety and Efficacy of Diquafosol Compared to Artificial Tears for the Treatment of Dry Eye: A Systematic Review and Meta-Analysis

**DOI:** 10.3390/ijms26178113

**Published:** 2025-08-22

**Authors:** José Gerardo Serrano-Robles, Ana Karen Pérez-Vázquez, Guillermo Raul Vera-Duarte, Alejandro Navas, Arturo Ramirez-Miranda, Enrique O. Graue-Hernandez, Nicolás Kahuam-López

**Affiliations:** 1 Instituto de Oftalmologia Fundacion Conde de Valenciana IAP, Mexico City 11520, Mexico; gerardoserranorobles@gmail.com (J.G.S.-R.);; 2 Centro de Investigación en Ciencias de La Salud Anáhuac (CICSA), Facultad de Ciencias de La Salud, Universidad Anáhuac México, Campus Norte, Mexico City 52786, Mexico

**Keywords:** systematic review, meta-analysis, secretagogues, artificial tears, dry eye, diquafosol

## Abstract

Dry eye disease (DED) is a prevalent and disabling condition. Artificial tears are commonly used but often inadequate for moderate-to-severe cases. Secretagogues such as pilocarpine, cevimeline, and diquafosol offer potential alternatives, though their comparative effectiveness remains unclear. To evaluate the safety and efficacy of these secretagogues versus artificial tears in adults with DED, we searched CENTRAL, PubMed, Scopus, LILACS, ClinicalTrials.gov, and WHO ICTRP without language restrictions. Randomized controlled trials (RCTs) comparing secretagogues to artificial tears were eligible. Data extraction and synthesis were conducted using Covidence and the Cochrane RoB 2 tool, and 19 RCTs (n = 2697) were included. Fifteen were analyzed quantitatively; however, only eight trials evaluating diquafosol were suitable for meta-analysis, as data for pilocarpine and cevimeline were insufficient for quantitative synthesis. GRADE was used to assess evidence certainty. PROSPERO registration: CRD42020218407. Diquafosol significantly improved rose bengal staining at 4 weeks and OSDI scores and TBUT in post-cataract patients at 4 and 12 weeks. However, it increased mild adverse events (RR, 1.81; 95% CI, 1.15–2.84). Evidence for pilocarpine and cevimeline was limited. Diquafosol 3% shows greater efficacy than artificial tears in post-cataract DED but with more side effects. Further research is needed for other secretagogues.

## 1. Introduction

Dry eye disease (DED) affects the ocular surface and is a leading cause of ophthalmological consultations [1,2]. The symptoms vary, ranging from a foreign body sensation to severe pain [3,4], disrupting daily activities, which can have a negative impact on the quality of life of patients. The Tear Film & Ocular Surface Society (TFOS) Dry Eye Workshop II (DEWS-II) defines DED as a multifactorial disease of the ocular surface characterized by a loss of homeostasis of the tear film accompanied by ocular symptoms, in which tear film instability and hyperosmolarity, ocular surface inflammation and damage, and neurosensory abnormalities play etiological roles [5]. It is estimated that the prevalence ranges from 5 to 35%, with female predominance and a maximum peak at age 60, where the prevalence reaches 70% with a more significant trend for the Asian population [3,4,6,7]. Different conditions that affect one or more components of the tear film or the glands that produce its components have the potential to cause the disease [4]. Tear hyperosmolarity is considered to be the trigger of a cascade of signaling events within corneal epithelial cells, leading to the release of inflammatory mediators and proteases [3]. 

The management of DED is complex due to its multifactorial etiology [8,9,10]. The treatment aims to restore homeostasis to the ocular surface and tear film, breaking the vicious cycle of the disease. Medical therapies for DED include tear replacement, anti-inflammatory medications, tear film retention, stimulation, and environmental modifications [11]. Topical treatment with artificial tears (ATs) is widely used in patients with dry eyes and can alleviate the signs and symptoms of patients with DED [12]. However, ATs alone may be insufficient to improve symptoms in some patients. In recent years, a scheme has been proposed for the treatment of DED [13], with artificial tears being the mainstay of treatment. While the use of secretagogues is suggested as part of the treatment for patients with moderate to severe degrees of the disease [14,15].

Various pharmacological agents with a secretagogue effect can stimulate watery secretion, mucus secretion, or both. Topical diquafosol eye drops have been favorably evaluated in several studies [6,16,17]. This agent can stimulate watery and mucous secretion in both animals and humans. It is also possible for the oral administration of cholinergic agonists, particularly pilocarpine and cevimeline, to treat severe DED. They have FDA-approved indications for the treatment of dry mouth associated with Sjögren’s syndrome (SS) [18]. Pilocarpine and cevimeline exert their therapeutic effect primarily through stimulation of muscarinic receptors on the lacrimal glands, enhancing aqueous tear secretion [19,20,21]. Although both are broadly classified as cholinergic agonists, their action is mainly mediated via muscarinic pathways, particularly the M3 subtype. Pilocarpine has broader activity across muscarinic receptors, while cevimeline shows greater selectivity for M3, which may result in more targeted glandular stimulation and fewer systemic effects. In contrast, diquafosol is a selective P2Y_2_ purinergic receptor agonist that promotes tear secretion through a distinct mechanism [22,23]. By activating P2Y_2_ receptors on the ocular surface epithelium, diquafosol stimulates chloride ion transport and mucin secretion from goblet cells, thereby contributing to both aqueous volume and tear film stability. Its local, non-cholinergic action may offer a more favorable tolerability profile and broader effects on the ocular surface environment compared to muscarinic agonists. Recent preclinical studies also suggest that formulations based on nanocarriers, such as gabapentin-loaded ceria nanoparticles combined with mucoadhesive polymers, can enhance ocular retention and promote not only tear secretion but also corneal nerve preservation and epithelial regeneration. In a rabbit model of dry eye, Yang et al. demonstrated that this approach significantly alleviated dry eye symptoms by increasing mucin-binding efficiency, prolonging ocular surface residence time, and exerting antioxidant and neuroprotective effects [24].

The objective of the current systematic review and meta-analysis is to determine the safety and efficacy of pilocarpine, cevimeline, and diquafosol compared to artificial tears for treating dry eye. However, due to limited data, only diquafosol was included in the quantitative synthesis, while the evidence for pilocarpine and cevimeline was assessed qualitatively.

## 2. Methods

### 2.1. Protocol and Registration

The review was registered on PROSPERO (registration number CRD42020218407) and was reported following the Preferred Reporting Items for Systematic Reviews and Meta-analyses (PRISMA) statement standard guidelines [25]. The methodology established in the published protocol [26] was followed during the systematic review.

### 2.2. Eligibility Criteria

We included randomized controlled trials in which the study population comprised adults aged 18 years or older with a clinical diagnosis of dry eye disease, including subtypes such as aqueous tear deficiency, Sjögren’s syndrome, or keratoconjunctivitis sicca. During the full-text review, we verified that the eligibility criteria of each trial met this specification, and when available, we reviewed the corresponding trial registry to confirm this information. Eligible interventions included treatment with pilocarpine, cevimeline, or diquafosol. The comparator was artificial tears. Studies were primarily conducted in outpatient settings, as dry eye disease does not typically require inpatient management. We imposed no restrictions based on language or publication status.

### 2.3. Databases and Information Sources

RCTs were searched in CENTRAL, PubMed, Scopus, LILACS, ClinicalTrials.gov, WHO, and ICTRP without language or date restrictions. Reference lists of included studies were also reviewed for additional trials.

### 2.4. Search Methods

A highly sensitive Cochrane strategy was used to identify RCTs, supplemented by the PRESS guideline [27,28]. Full search strategies for each database are available in Supplementary Materials S1.

### 2.5. Study Eligibility Criteria

Two reviewers (GSR and AKP) independently performed a study assessment following a standardized approach. Any reviewer disagreement was settled by discussion or consulting a third review author if required (NKL). We followed the criteria of inclusion, exclusion, and elimination established in the published protocol [26]. 

### 2.6. Outcome Measures

Evaluated changes in dry eye signs (TBUT, rose bengal and fluorescein staining, Schirmer test) and quality of life (VRQoL or OSDI). Adverse events (e.g., irritation, pain, conjunctivitis) were also assessed. Data from all reported time points were extracted, and a stratified analysis was performed using trials with matching time points.

### 2.7. Shared Time Points and Outcomes

Information regarding shared outcomes and evaluated time points across studies is available in the Supplementary Material. This includes a detailed summary of randomized controlled trials comparing diquafosol, with and without a history of cataract surgery, as well as the specific time points assessed in each study, even in cases where outcomes were comparable but time points differed.

### 2.8. Data Collection and Analysis

**Figure 1:**
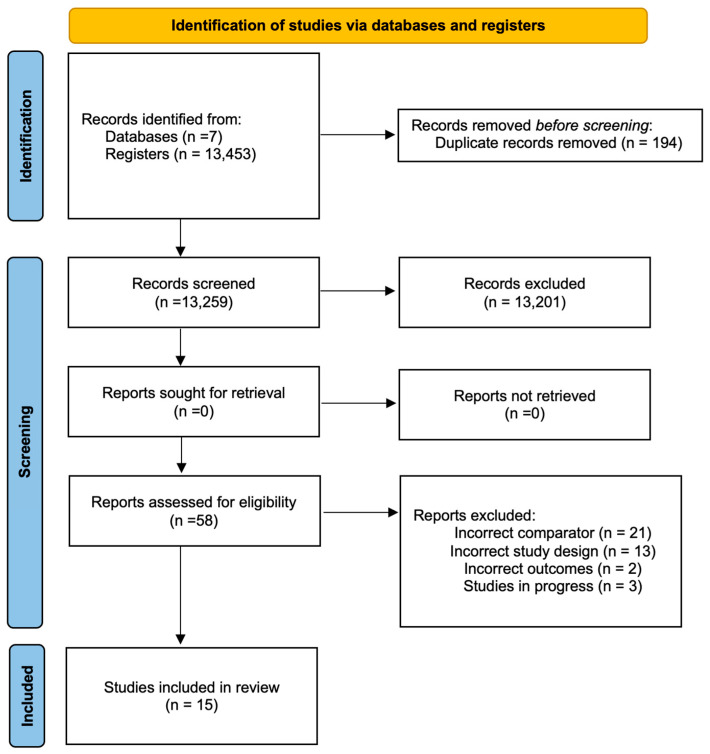
PRISMA flowchart summarizing study selection for the systematic review and meta-analysis.

Study selection was independently performed by two reviewers using Covidence [29], with disagreements resolved by a third author. Titles and abstracts were screened, followed by full-text assessment of potentially relevant studies. Reasons for exclusions were recorded, and the selection process was presented in a PRISMA diagram (Figure 1). 

### 2.9. Risk of Bias Assessment

The Risk of Bias 2 (RoB 2) tool from the Cochrane Collaboration was used to evaluate bias across key domains. Two reviewers independently assessed each study, classifying risk as “low, high,” or “unclear.” Discrepancies were resolved by a third reviewer.

### 2.10. Statistical Analysis

Meta-analyses were conducted using RevMan 5.3. Mean differences (MD) with 95% confidence intervals were calculated for continuous outcomes, and relative risks were used for dichotomous outcomes. Heterogeneity was assessed via forest plots, Chi-square test, and I^2^ statistic, following Cochrane guidelines. WebPlotDigitizer was used when outcome data were only available in graphical format.

## 3. Results

### 3.1. Literature Search

From 13,453 records, 194 duplicates were removed, and 13,259 titles/abstracts were screened. A total of 58 articles underwent full-text review; 39 were excluded for reasons including incorrect comparator (21), study design (13), outcomes (2), or ongoing status (3). One pilocarpine trial was excluded from meta-analysis due to being a single study. Three cevimeline trials were identified, but none were included in the meta-analysis due to lack of shared outcomes or design heterogeneity. Ultimately, 19 studies were included in the qualitative synthesis and 15 in the meta-analysis. Full study selection details are presented in Figure 1.

#### Risk-of-Bias Assessment

Supplementary Materials S2 presents bias risk summaries for the trials included in this review. In the comparison between Diquafosol and artificial tears, it is noted that four clinical trials exhibited low bias risk, while four raised some concerns. Potential bias sources were identified across all domains except the outcome selection domain, indicating a 50% overall low bias risk and 50% of trials with some source of concern.

A detailed visual summary of the qualitative synthesis is provided in the Supplementary Material (Supplementary Materials S2).

### 3.2. Interventions

#### 3.2.1. Diquafosol 3% vs. Artificial Tears

All included trials compared Diquafosol 3% with artificial tears. Some focused on post-cataract patients and were analyzed separately due to their specific clinical context. Most were conducted in outpatient ophthalmology settings. Only studies reporting identical outcomes at the same time points were included in the quantitative synthesis.

Several trials assessed TBUT at 2 weeks [30,31,32] and 4 weeks [30,31,32,33] post-treatment. Fluorescein staining was evaluated at 2 [30,31,32,33,34] and 4 weeks [30,31,32,33,34], while rose bengal staining was reported at 2 [30,32,34] and 4 weeks [30,32,33,34].

#### 3.2.2. Diquafosol 3% in Post-Cataract Patients

Two trials reported OSDI scores at 1, 4, and 12 weeks post-surgery [16,35]. TBUT was assessed at 1 [16,35], 4 [16,35,36,37,38,39], and 12 weeks [16,35,38]. Four studies reported STT results across 1, 4, and 12 weeks [16,35,36,38], with Jun et al. [38] focusing on 4 and 12 weeks, and Inoue et al. [36] only at 4 weeks. Fluorescein staining was reported at 4 [36,37,38] and 12 weeks [16,38].

#### 3.2.3. Pilocarpine vs. Artificial Tears

One RCT by Tsifetaki et al. [40] evaluated pilocarpine in 85 subjects randomized to receive pilocarpine, artificial tears, or punctal occlusion. Outcomes included rose bengal and fluorescein staining over 12 weeks. Due to being a single study, it was excluded from meta-analysis.

#### 3.2.4. Cevimeline Trials

Three RCTs evaluated cevimeline in Sjögren’s patients. Petrone et al. [41] included 197 subjects; only the 30 mg TID group was analyzed, with STT measured over 12 weeks. Ono et al. [18] evaluated TBUT, STT, and staining at 2 and 4 weeks. Leung et al. [42] conducted a crossover trial with a 4-week washout, but extractable outcomes were unavailable. Quantitative synthesis was not feasible due to heterogeneous endpoints, though all studies reported favorable effects.

### 3.3. Effects of Interventions

#### 3.3.1. Diquafosol 3% vs. Artificial Tears

**Figure 2:**
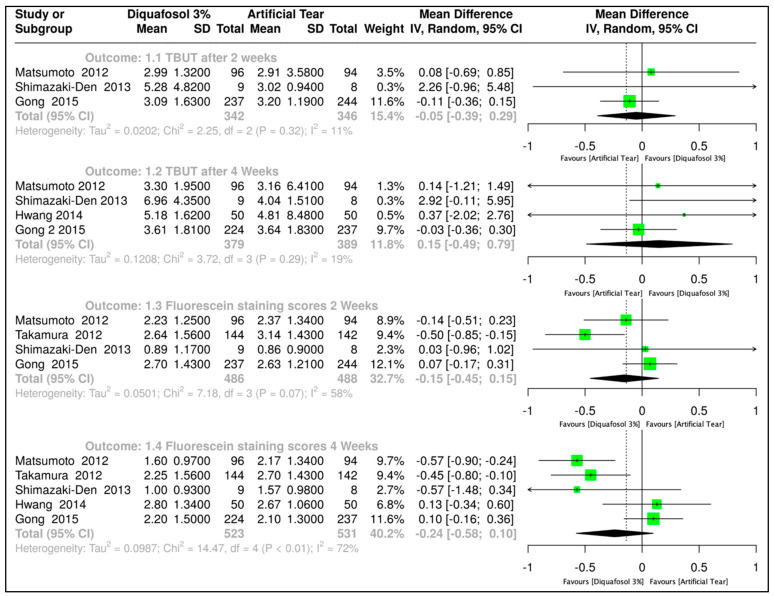
Forest plot comparing 3% diquafosol versus artificial tears for tear breakup time (TBUT) and corneal fluorescein staining. Results are presented as mean differences with 95% confidence intervals (CIs), based on data from studies by Matsumoto et al. [30], Shimazaki-Den et al. [31], Gong et al. [32], Hwang et al. [33], and Takamura et al. [34].

TBUT is a continuous quantitative outcome measured in seconds. Thus, an MD analysis with a random-effects model was employed, calculating a 95% confidence interval (CI). Four clinical trials evaluated the effect after two weeks of treatment [30,31,32,33]. The combined data from all trials resulted in 342 subjects assigned to the Diquafosol 3% treatment and 346 subjects to artificial tears (MD, −0.05, 95% CI, −0.39 to 0.29; Figure 2). Similarly, four trials assessed the effect after four weeks of treatment [30,31,32,33]. The combined data from all trials resulted in 379 subjects assigned to the Diquafosol 3% treatment and 389 subjects to artificial tears (MD, 0.15 with 95% CI, −0.49 to 0.79; Figure 2). In both cases, the *p*-value was greater than 0.05, indicating no statistically significant difference between using Diquafosol 3% and artificial tears for this outcome.

Fluorescein stain score was assessed by MD analysis with a random-effects model employed, with 95% CI. Four clinical trials evaluated the effect of Diquafosol 3% versus artificial tears after two weeks of treatment [30,31,32,34]. The combined data from all trials resulted in 486 subjects assigned to Diquafosol 3% and 488 subjects assigned to artificial tears treatment (MD, −0.15, 95% CI, −0.45 to 0.15; Figure 2). Five trials assessed the effect of Diquafosol 3% versus artificial tears after four weeks of treatment [30,31,32,33,34]. The combined data from all trials resulted in 523 subjects assigned to Diquafosol 3% and 531 subjects assigned to artificial tears treatment, yielding an MD of −0.24, 95% CI, −0.58 to 0.10 (Figure 2). In both cases, the *p*-value was greater than 0.05, indicating no statistically significant difference between using Diquafosol 3% and artificial tears for this outcome.

#### 3.3.2. Diquafosol 3% vs. Artificial Tears After Cataract Surgery

**Figure 3:**
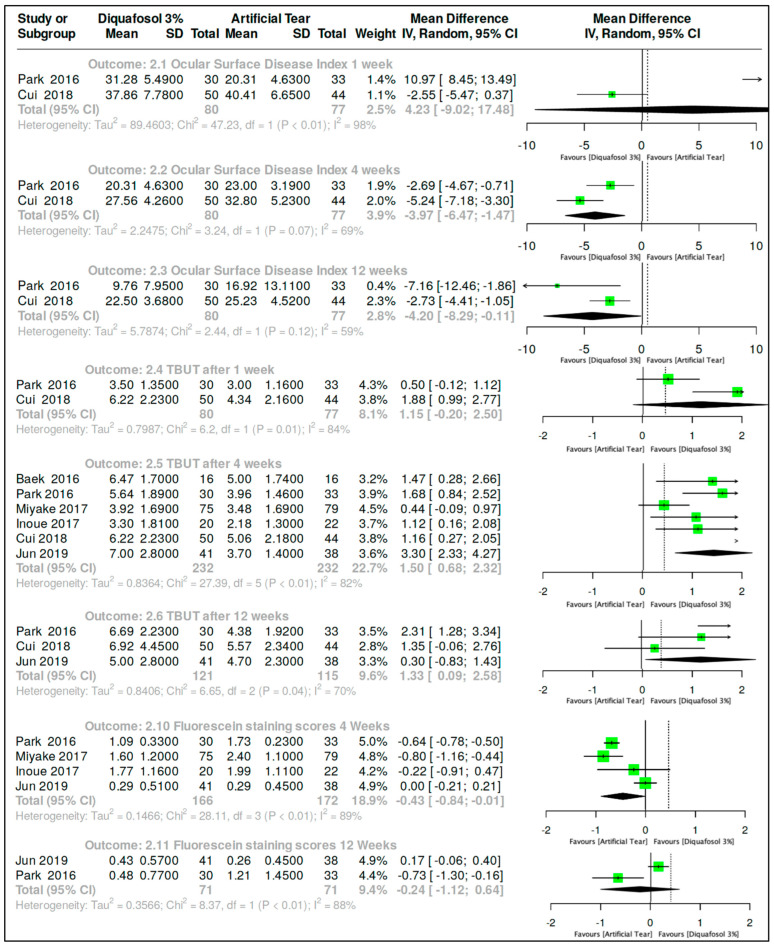
Forest plot comparing 3% diquafosol versus artificial tears in post-cataract surgery patients, evaluating Ocular Surface Disease Index (OSDI), tear breakup time (TBUT), and corneal staining. Results are shown as mean differences with 95% confidence intervals (CIs), based on studies by Park et al. [16], Cui et al. [35], Baek et al. [39], Miyake et al. [37], Inoue et al. [36], and Jun et al. [38].

OSDI outcome was assessed by MD analysis with a random-effects model performed for this outcome. Two clinical trials [16,35] evaluated the effect of Diquafosol 3% versus artificial tears in subjects post-cataract surgery after one week of treatment. The combined data from these trials resulted in 80 subjects assigned to Diquafosol 3% and 77 subjects assigned to the artificial tears treatment (yielding MD, 4.23, 95% CI, −9.02 to 17.48; Figure 3). Both trials assessed the outcome at four and twelve weeks of treatment, resulting in MD, −3.97.95% CI, −6.47 to −1.47 and −4.20, 95% CI, −8.29 to −0.11, respectively (Figure 3). In the one-week comparison, there was no difference between the use of Diquafosol 3% and artificial tears in subjects post-cataract surgery (*p* > 0.05). However, in the four-week and twelve-week comparisons, the results were, respectively, Z = 3.12, *p*-value of 0.002, and Z = 2.01, *p*-value of 0.04, showing a better response in the Diquafosol 3% group.

TBUT is a continuous quantitative outcome; thus, MD analysis with a random-effects model was utilized, 95% CI. Two clinical trials [16,35] evaluated the effect of Diquafosol 3% versus artificial tears in subjects post-cataract surgery after one week of treatment. The combined data from these trials resulted in 80 subjects assigned to Diquafosol 3% and 77 subjects assigned to the artificial tears treatment, yielding MD 1.15, 95% CI, −0.20 to 2.50 (Figure 3). It was also possible to compare this outcome at four weeks of treatment since six clinical trials reported the outcome [16,35,36,37,38,39]. The combined data from these trials resulted in 232 subjects assigned to Diquafosol 3% and 232 subjects assigned to the artificial tears treatment, yielding MD 1.50, 95% CI, 0.68 to 2.32 (Figure 3). A prediction interval was also calculated for this outcome, with 95% of the data falling between −0.18 and 3.18.

The outcome was also evaluated at 12 weeks in three studies [16,35,38]. Combined data from these trials resulted in 121 subjects assigned to Diquafosol 3% and 115 subjects assigned to the artificial tears treatment, yielding an MD of 1.33, 95% CI, 0.09 to 2.58 (*p* > 0.05), in the 1-week comparison (Figure 3). However, in the 4-week and 12-week comparisons, the results were, respectively, Z = 3.58, *p*-value of 0.0003 and Z = 2.10, *p*-value of 0.04, showing a better response in the Diquafosol 3% group.

Fluorescein stain score is a continuous quantitative outcome; hence, MD analysis with a random-effects model was used. Four clinical trials evaluated the effect of Diquafosol 3% versus artificial tears in subjects post-cataract surgery after one week of treatment [16,36,37,38]. The combined data from these trials resulted in 166 subjects assigned to Diquafosol 3% and 172 subjects assigned to the artificial tears treatment, yielding an MD −0.43 with a 95% CI, −0.84 to −0.01 (Figure 3). It was also possible to compare this outcome at twelve weeks of treatment since two clinical trials reported the outcome [16,38]. The combined data from these trials resulted in 71 subjects assigned to Diquafosol 3% and 71 subjects assigned to the artificial tears treatment, yielding an MD of −0.24 with a 95% CI, −1.12 to 0.64 (Figure 3). The Z = 2.01, *p*-value 0.04 in the 4-week comparison, showing a better response in the Diquafosol 3% group. However, in the 12-week comparison, the *p*-value was 0.59, indicating no statistically significant difference between the use of Diquafosol 3% and artificial tears in subjects post-cataract surgery at this time point.

### 3.4. Safety Outcomes

Diquafosol 3% vs. Artificial Tears

**Figure 4:**
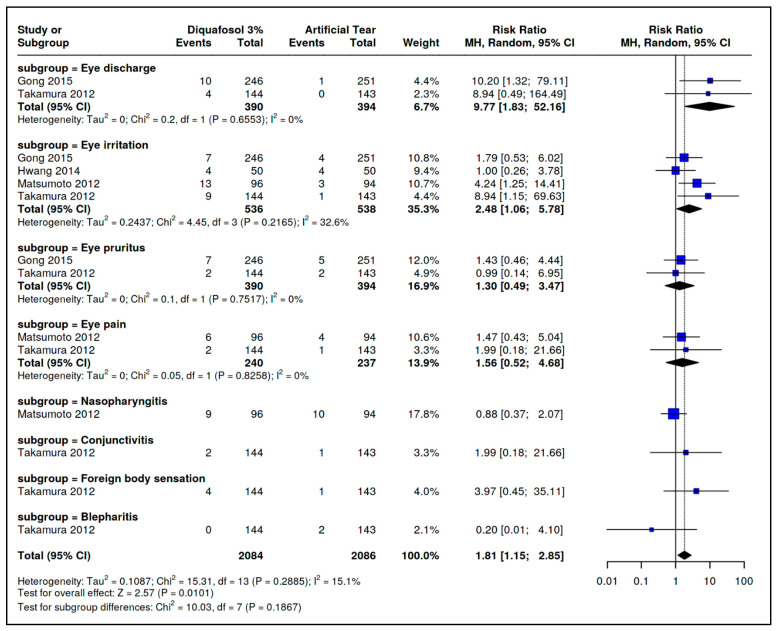
Forest plot comparing the incidence of adverse events between 3% diquafosol and artificial tears. Results are reported as risk ratios with 95% confidence intervals (CIs), based on data from Gong et al. [32], Takamura et al. [34], Hwang et al. [33], and Matsumoto et al. [30].

Ocular Secretion: Two trials [32,34] reported ocular secretion. Pooled data showed a higher risk in the Diquafosol 3% group (RR = 9.77; 95% CI: 1.83–52.16; I^2^ = 0%; Figure 4).

Ocular Irritation: Four trials [30,32,33,34] reported ocular irritation, with increased risk in the Diquafosol group (RR = 2.48; 95% CI: 1.06–5.78; I^2^ = 33%; Figure 4).

Ocular Itching: This was reported in two trials [32,34], with no significant difference between groups (RR = 1.30; 95% CI: 0.49–3.47; I^2^ = 0%; Figure 4).

Ocular Pain: This was also reported in two trials [32,34], showing no significant difference (RR = 1.56; 95% CI: 0.52–4.68; I^2^ = 0%; Figure 4).

Nasopharyngitis: This was reported by Matsumoto et al. [30] (RR = 0.88; 95% CI: 0.37–2.07). Only one trial reported this outcome, so I^2^ was not estimated (Figure 4).

Conjunctivitis: This was reported by Takamura et al. [34] (RR = 1.99; 95% CI: 0.18–21.66). I^2^ could not be calculated (Figure 4).

Foreign Body Sensation: Takamura et al. [34] reported this event with higher risk in the artificial tears group (RR = 3.97; 95% CI: 0.08–4.10; Figure 4).

Blepharitis: This was also reported by Takamura et al. [34], favoring Diquafosol (RR = 0.20; 95% CI: 0.01–4.10; Figure 4).

Meta-Analytic Result: Overall, Diquafosol 3% was associated with a higher risk of adverse events (RR = 1.81; 95% CI: 1.15–2.84; Figure 4).

## 4. Discussion

This systematic review included only clinical trials assessing the safety and efficacy of pilocarpine, cevimeline, and diquafosol versus artificial tears for dry eye treatment. For pilocarpine (Tsifetak et al. [40]) and cevimeline (three trials: [18,41,42]), quantitative synthesis was not feasible due to a lack of shared outcomes or aligned time points; thus, only qualitative analysis was performed.

Eight clinical trials comparing Diquafosol to artificial tears were identified. A stratified analysis included only studies reporting the same outcomes at the same time points. For TBUT at 2 and 4 weeks, four trials [30,31,32,33] involving 768 participants (379 Diquafosol and 389 artificial tears) found no statistically significant difference. For fluorescein staining, four trials [30,31,32,34] at 2 weeks (n = 974) and five trials [30,31,32,33,34] at 4 weeks (n = 1054) also showed no significant difference.

In post-cataract surgery patients, seven trials were included. Two trials [16,35] assessed OSDI in 157 patients; no difference was found at week 1, but Diquafosol was significantly superior at weeks 4 and 12 (MDs −3.97 and −4.20; I^2^ < 70%, high certainty, Table 1). For TBUT, six trials [16,35,36,37,38,39] with 464 subjects showed a significant benefit at 4 weeks (MD 1.5), with moderate certainty.

Table 1 summarizes adverse effects. Diquafosol 3% was associated with a higher incidence of symptoms such as discharge, irritation, itching, and conjunctivitis. The evidence indicated a significantly increased risk, with moderate certainty despite some imprecision.

### 4.1. Quality of the Evidence

Only randomized clinical trials were included. Risk of bias was independently assessed using Cochrane’s RoB 2 tool, covering five domains: randomization, intervention deviations, missing data, outcome measurement, and selective reporting.

For Diquafosol vs. artificial tears, four studies were rated low risk [30,43,44,45], while four had moderate risk [31,32,33,34], primarily due to randomization and measurement issues. Among post-cataract trials, three were low risk [35,38,46] and three moderate risk [16,39,45]; Inoue et al. [36] was rated high risk due to incomplete data.

Regarding evidence certainty, two outcomes had moderate certainty and four had low certainty in the Diquafosol–artificial tears comparison, mainly downgraded for reliance on graphical data and subjective endpoints. In post-cataract studies, two outcomes had high certainty, one moderate certainty, six low certainty, and one very low certainty, affected by inconsistency, imprecision, and graphical-only reporting, particularly for fluorescein staining. Despite standardized grading, subjectivity in rose bengal scoring may have influenced results. For adverse events, one outcome had high certainty and four had moderate certainty. Ocular secretion was upgraded to high due to strong and consistent effects (RR > 2.0 or <0.5) across studies. 

Although a previous meta-analysis by Liu et al. [47] addressed the effects of 3% diquafosol in dry eye disease, our study differs in several important aspects. We conducted a broader and more sensitive literature search, included a larger number of randomized controlled trials, and focused specifically on comparisons with artificial tears. Our review also considered other topical secretagogues such as pilocarpine and cevimeline; while meta-analysis was not feasible for these agents, the search strategy retrieved relevant studies that were narratively reviewed. In addition, we performed a subgroup analysis in post-cataract surgery patients and assessed methodological quality using Cochrane RoB 2.0 and GRADE criteria. For adverse events, one outcome had high and four had moderate certainty; notably, ocular secretion was upgraded to high due to strong and consistent effects (RR > 2.0 or <0.5) across studies. The protocol was prospectively registered and published, ensuring methodological transparency. Xinyu Zhao et al. [48] also conducted a prior meta-analysis on diquafosol in post-cataract patients, reporting favorable outcomes; however, their review lacked protocol registration, risk of bias assessment, and a defined search strategy, which limits its reliability. Nevertheless, their findings are consistent with ours.

This is the first systematic review specifically evaluating 3% Diquafosol vs. artificial tears, showing greater benefits in post-cataract patients likely due to enhanced action in surgically altered ocular surfaces, compared to its tear-like function in non-surgical eyes. A major limitation was the absence of data on tear osmolarity or vision-related quality of life (VR-QoL), despite being pre-specified outcomes. Inconsistent follow-up time points across trials also reduced comparability, limiting both the quantity and certainty of the evidence. Furthermore, the composition of artificial tears varied across the included trials. Some studies used carboxymethylcellulose-based formulations, others used sodium hyaluronate at different concentrations, and several did not specify the exact components of the comparator. These differences may have influenced tear film retention time, osmolarity, and surface coverage, introducing variability in the comparator group and potentially affecting treatment outcomes. We now highlight this formulation heterogeneity as a limitation that may reduce the internal consistency of the control arm. Future trials should clearly specify and, where possible, standardize the artificial tear formulation to improve comparability across studies.

### 4.2. Overall Completeness and Applicability of Evidence

The clinical trials included in this systematic review were not substantially heterogeneous in terms of population or interventions. However, the available evidence was insufficient to comprehensively address all predefined objectives of the review. Key patient-centered outcomes, such as vision-related quality of life (VRQoL) and tear osmolarity, were not reported in the included studies. Additionally, relevant measures such as intraoperative or postoperative discomfort, treatment adherence, and long-term safety were either inconsistently reported or entirely absent. For pilocarpine and cevimeline, the effects of different doses on both efficacy and adverse events were not systematically explored. Moreover, the potential differences in effectiveness and tolerability between systemic (oral) and topical ophthalmic administration remain unclear, as direct comparisons are lacking. Considering that dry eye disease is driven by tear hyperosmolarity and ocular surface inflammation, future studies and meta-analyses should also incorporate relevant biological markers, such as tear osmolarity, inflammatory cytokine levels, or matrix metalloproteinase activity, to better understand the mechanisms and therapeutic effects of secretagogues. Importantly, dry eye disease encompasses multiple clinical subtypes, including aqueous-deficient and evaporative forms, which may respond differently to treatment. A more granular classification of patients according to disease subtype is needed to accurately assess treatment efficacy and to guide personalized therapy. These limitations restrict the clinical applicability of the current findings and underscore the need for more comprehensive, stratified, and standardized outcome reporting in future randomized trials.

## 5. Conclusions

Based on the findings of this systematic review and meta-analysis, 3% diquafosol demonstrated superiority over artificial tears in improving rose bengal staining after four weeks of treatment. In post-cataract surgery patients, diquafosol was also superior in improving OSDI and TBUT at four and twelve weeks, and STT at one, four, and twelve weeks. Additionally, fewer adverse effects were reported with diquafosol in some trials. These benefits were supported by moderate- to high-certainty evidence in postoperative populations, suggesting that diquafosol may be considered a more effective treatment option than artificial tears in this subgroup. However, in other populations with dry eye disease, findings were inconsistent and based on low- to very low-certainty evidence, limiting generalizability. Regarding pilocarpine and cevimeline, the current evidence is insufficient to support clinical recommendations due to heterogeneity, limited trial data, and lack of shared outcomes. Therefore, clinical decisions should be made on a case-by-case basis, considering patient characteristics and disease subtype, with diquafosol reserved for specific contexts where evidence supports its use.

### Implications for Research

There is a clear need for further well-designed randomized controlled trials evaluating the safety and efficacy of secretagogues in diverse dry eye populations. Future studies should focus on the following:

- Compare treatment outcomes across clinically distinct subtypes of dry eye disease (e.g., aqueous-deficient vs. evaporative);
- Assess the dose–response relationships and adverse event profiles of pilocarpine and cevimeline;
- Investigate potential differences in efficacy and tolerability between systemic (oral) and topical administration routes;
- Incorporate relevant biological indicators (e.g., tear osmolarity, inflammatory cytokines, MMP-9) to better understand mechanisms of action;
- Include validated patient-centered outcomes such as vision-related quality of life and standardized symptom questionnaires;
- Prospectively register protocols and follow predefined methods and outcomes;
- Clearly describe randomization, allocation concealment, and masking procedures;
- Document handling of missing data and perform intention-to-treat analyses;
- Evaluate outcomes at standardized time points (e.g., 4, 12, and 24 weeks);
- Report adverse events in a stratified and comprehensive manner.

Finally, future trials should adopt a more granular classification of participants according to dry eye subtype and disease severity. This stratification, along with improved methodological consistency, will enhance the applicability of findings and inform evidence-based, personalized treatment strategies in dry eye disease.

## Figures and Tables

**Table 1:** Summary (GRADE) for the comparison of Diquafosol 3% vs. artificial tears in dry eye disease.

Comparison Diquafosol 3% vs. Artificial Tears									
Certainty Assessment					No. of Patients		Effect Size		Certainty
No Studies	Study Design	Risk of Bias	Inconsistency	Imprecision	[Diquafosol 3%]	[Artificial Tear]	Absolute (95% CI)		
Tear Film Breakup Time after two weeks of treatment									
3	Randomized trials	Not serious	Not serious	Serious ^a^	342	346	MD 0.05 (0.39–0.29)		⨁⨁⨁◯ Moderate
Tear Film Breakup Time after four weeks of treatment									
4	Randomized trials	Not serious	Not serious	Serious ^a^	379	389	MD 0.15 (0.49–0.79)		⨁⨁⨁◯ Moderate
Fluorescein staining score two weeks of treatment									
4	Randomized trials	Not serious	Serious ^b^	Serious ^a^	486	488	MD 0.15 (0.45–0.15)		⨁⨁◯◯ Low
Fluorescein staining score four weeks of treatment									
5	Randomized trials	Not serious	Serious ^b^	Serious ^a^	523	531	MD 0.24 (0.58–0.1)		⨁⨁◯◯ Low
Comparison Diquafosol 3% vs. Artificial Tears in post-cataract surgery subjects									
Ocular Surface Disease Index after one week of treatment.									
2	Randomized trials	Not serious	Serious ^c^	Serious ^d^	80	77	MD 4.23 (9.02–17.48)		⨁⨁◯◯ Low
Ocular Surface Disease Index after four weeks of treatment.									
2	Randomized trials	Not serious	Not serious	Not serious	80	77	MD 3.97 (6.47–1.47)		⨁⨁⨁◯ Moderate
Ocular Surface Disease Index after twelve weeks of treatment.									
2	Randomized trials	Not serious	Not serious	Not serious	80	77	MD 4.2 (8.29–0.11)		⨁⨁⨁◯ Moderate
Tear Film Breakup Time after one week of treatment.									
2	Randomized trials	Not serious	Serious ^e^	Serious ^e^	80	77	MD 1.15 (0.2–2.5)		⨁⨁◯◯ Low
Tear Film Breakup Time after four weeks of treatment.									
6	Randomized trials	Serious ^c^	Not serious	Not serious	232	232	MD 1.5 (0.68–2.32)		⨁⨁⨁◯ Moderate
Tear Film Breakup Time after twelve weeks of treatment.									
3	Randomized trials	Not serious	Serious ^c^	Serious ^e^	121	115	MD 1.33 (0.09–2.58)		⨁⨁◯◯ Low
Fluorescein staining after four weeks of treatment.									
4	Randomized trials	Serious ^e^	Serious ^e^	Serious ^b^	166	172	MD 0.43 (0.84–0.01)		⨁◯◯◯ Very low
Fluorescein staining after four weeks of treatment.									
2	Randomized trials	Not serious	Serious ^e^	Serious ^b^	71	71	MD 0.24 (1.12–0.64)		⨁⨁◯◯ Low
Adverse Effects of the Comparison Diquafosol 3% vs. Artificial Tears									
Certainty Assessment					No. of Patients		Effect Size		Certainty
No Studies	Study Design	Inconsistency	Imprecision	Other Considerations	[Diquafosol 3%]	[Artificial Tear]	Relative (95% CI)	Absolute (95% CI)	
Adverse event: Ocular discharge									
2	Randomized trials	Not serious	Serious ^a^	Strong association	14/390 (3.6%)	1/394 (0.3%)	RR 9.77 (1.83–52.16)	22 more per 1000 (from 2 more to 130 more)	⨁⨁⨁◯ Moderate
Adverse event: Eye Irritation									
4	Randomized trials	Serious ^c^	Serious ^a^	Strong association	33/536 (6.2%)	12/538 (2.2%)	RR 2.48 (1.06–5.78)	33 more per 1000 (from 1 more to 107 more)	⨁⨁⨁◯ Moderate
Adverse event: Ocular itching									
2	Randomized trials	Not serious	Serious ^a^	None	9/390 (2.3%)	7/394 (1.8%)	RR 1.30 (0.49–3.47)	5 more per 1000 (from 9 less to 44 more)	⨁⨁⨁◯ Moderate
Adverse event: Eye pain									
2	Randomized trials	Not serious	Serious ^a^	None	8/240 (3.3%)	5/237 (2.1%)	RR 1.56 (0.52–4.68)	12 more per 1000 (from 10 less to 78 more)	⨁⨁⨁◯ Moderate
Adverse event: Conjunctivitis									
1	Randomized trials	Not serious	Serious ^a^	None	2/144 (1.4%)	1/143 (0.7%)	RR 1.99 (0.18–21.66)	7 more per 1000 (from 6 less to 144 more)	⨁⨁⨁◯ Moderate
Adverse event: Foreign body sensation									
1	Randomized trials	Not serious	Serious ^a^	None	4/144 (2.8%)	1/143 (0.7%)	RR 3.97 (0.45–5.11)	7 more per 1000 (from 6 less to 144 more)	⨁⨁⨁◯ Moderate
Adverse event: Blepharitis									
1	Randomized trials	Not serious	Serious ^a^	None	0/144 (0.0%)	2/143 (1.4%)	RR 0.20 (0.01–4.10)	11 less per 1000 (from 14 less to 43 more)	⨁⨁⨁◯ Moderate

CI: confidence interval; MD: mean difference; RR: relative risk. ^a^ Some trials reported information on this outcome in graphic form, so data extraction software was used, with the possibility of introducing variation. ^b^ Although all clinical trials used the same method to score this outcome, the perception of each rater may influence the scoring of the reported results. ^c^ Conflicting evidence reported in clinical trials; ^d^ inaccurate evidence; ^e^ some clinical trials included in this comparison had a high risk of bias. Moderate certainty: the estimated effect is probably close to the true effect, although a meaningful difference cannot be ruled out. Low certainty: confidence in the estimate is limited, and the actual effect could differ significantly. Very low certainty: there is minimal confidence in the estimate; the true effect is likely to be markedly different.

## Data Availability

All data supporting the findings of this study are available in the Supplementary Materials provided with the manuscript. No new primary data were generated during this systematic review and meta-analysis, as all analyses are based on data extracted from previously published studies.

## Supplementary Materials

The following supporting information can be downloaded at: https://www.mdpi.com/article/10.3390/ijms26178113/s1.
