# Supplementary material for: Safety and Efficacy of Diquafosol Compared to Artificial Tears for the Treatment of Dry Eye: A Systematic Review and Meta-Analysis
Source: Int J Mol Sci. 2025 Aug 22;26(17):8113. doi: 10.3390/ijms26178113 (PMC12428352; doi:10.3390/ijms26178113)
Supplement: Supplementary file 1 [file ijms-26-08113-s001.zip › ijms-3689924-supplementary.pdf]

Supplementary Materials S1

PubMed
#1 *((randomized controlled trial[pt]) OR (controlled clinical trial[pt]) OR (randomized[tiab] OR randomized[tiab]) OR (randomly[tiab]) OR (trial[tiab]) OR (groups[tiab])) NOT (animals[mh] NOT humans[mh]).*
#2 *(Dry eye [tw] OR Dry eye syndrome [tw] OR Dry eye disease [tw] OR Conjunctivitis sicca [tw] OR Keratoconjunctivitis sicca [tw] OR Keratitis sicca [tw]).*
#3 *(Pilocarpine [pa] OR Cevimeline [pa] OR Diquafosol [pa]) OR (Pilocarpine [tiab] OR Cevimeline [tiab] OR Diquafosol [tiab]).*
#4 *(Artificial tear [tiab] OR Ocular lubricant [tiab]) OR (Artificial tear [pa] OR Ocular lubricant [pa]).*
#5 *#1 AND #2 OR #3 OR #OR*

Cochrane Central Register of Controlled Trials (CENTRAL).

#1 *MeSH descriptor: [Dry Eye] explode all trees.*
#2 *MeSH descriptor: [Dry Eye Disease] explode all trees.*
#3 *MeSH descriptor: [Dry Eye Syndrome] explode all trees.*
#4 *(Pilocarpine):kw.*
#5 *(Cevimeline):kw.*
#6 *(Diquafosol):kw.*
#7 *(Artificial tear):kw.*
#8 *(Ocular lubricant):kw.*
#9 *#1 OR #2 OR #3 OR #4 OR #5 #6 OR #7 OR #8*

LILACS
(TW:”randomized controlled trial” OR TW:”controlled clinical trial” OR TW:randomized OR TW:randomized OR TW:randomly OR TW:trial OR TW:groups) AND ((TW:”Dry eye” OR TW:”Dry eye syndrome” OR TW:”Dry eye disease” OR TW:”Conjunctivitis sicca” OR TW:”Keratoconjunctivitis sicca” OR TW:”Keratitis sicca”) OR (TW:Pilocarpine OR TW:Cevimeline OR TW:Diquafosol) OR (TW:”Artificial tear” OR TW:”Ocular lubricant”)).

ClinicalTrials.gov
((Dry eye OR Dry eye syndrome OR Dry eye disease OR Conjunctivitis sicca OR Keratoconjunctivitis sicca OR Keratitis sicca) OR (Pilocarpine OR Cevimeline OR Diquafosol) OR (Artificial tear OR Ocular lubricant)).

WHO ICTRP
((Dry eye OR Dry eye syndrome OR Dry eye disease OR Conjunctivitis sicca OR Keratoconjunctivitis sicca OR Keratitis sicca) OR (Pilocarpine OR Cevimeline OR Diquafosol) OR (Artificial tear OR Ocular lubricant)).

Scopus

((randomized AND controlled AND trial) OR (controlled AND clinical AND trial) OR (randomized OR randomized) OR (randomly) OR (trial) OR (groups)) AND (dry AND eye OR dry AND eye AND syndrome OR dry AND eye AND disease OR conjunctivitis AND sicca OR keratoconjunctivitis AND sicca OR keratitis AND sicca) AND (pilocarpine OR cevimeline OR diquafosol) OR (artificial AND tear OR ocular AND lubricant).

Databases and information sources

The following electronic databases for randomized controlled trials were searched. There were no language or publication year restrictions.

• Cochrane Central Register of Controlled Trials (CENTRAL) (which contains the CEV Trials Register) in the Cochrane Library (latest issue)
• PubMed (1948–2024)
• Scopus (2004–2024)
• LILACS (Latin American and Caribbean Health Sciences Information Database (1982–2024)
• US National Institutes of Health Ongoing Trials Register ClinicalTrials.gov
• World Health Organization (WHO)
• International Clinical Trials Registry Platform (ICTRP)

Study eligibility criteria

Two reviewers (GSR and AKP) independently performed a study assessment following a standardized approach. Any reviewer disagreement was settled by discussion or consulting a third review author if required (NKL). We followed the criteria of inclusion, exclusion, and elimination established in the published protocol [11].

Outcome measures

Change in dry eye disease signs, quantified by:
◦ Change in tear film stability, (TBUT)
◦ Change in the staining of the ocular surface (rose bengal stain score)
◦ Change in the staining of the ocular surface (fluorescein stain score)
◦ Change in the production of aqueous tears, (STT)
◦ Change in Quality of life, evaluated by the VRQoL Score (Vision-Related Quality of Life) or OSDI.

The incidence of adverse outcomes between treatment groups at various intervals.

We searched and extracted information from all the time points reported in the clinical trials; then we did a stratified analysis combining only information from clinical trials that shared the same time points. Adverse events were evaluated based on the data provided by the included studies. We specifically focused on adverse events such as eye discharge, eye irritation, pruritus, eye pain, conjunctivitis, foreign body sensation, blepharitis, and allergic conjunctivitis.

Comparison RCT including Cevimeline

Studies included: Petrone (2002), Ono (2004), Leung (2008)

- Evaluated time points: All included baseline measurement
  - Petrone (2002): 3 weeks, 6 weeks, 9 weeks, 12 weeks
  - Ono (2004): 2 weeks, 4 weeks
  - Leung (2008): 2 weeks, 6 weeks, 10 weeks

Shared time points and outcomes:

Petrone (2002) and Ono (2004) shared the STT outcome but did not share time points.

Comparison RCT including Diquafasol Without Cataract History

Studies included: Takamura (2012), Matsumoto (2012), Shimazaki-Den (2013), Hwang (2014), Gong (2015), Miyake H. (2016), Kaido (2018), Fukuoka (2019)

- Evaluated time points: All included baseline measurement
  - Takamura (2012): 2 weeks, 4 weeks
  - Matsumoto (2012): 2 weeks, 4 weeks, 6 weeks
  - Shimazaki-Den (2013): 2 weeks, 4 weeks
  - Hwang (2014): 4 weeks, 8 weeks, 12 weeks
  - Gong (2015): 2 weeks, 4 weeks
  - Miyake H. (2016): 10 min, 220 min
  - Kaido (2018): 5 weeks
  - Fukuoka (2019): 30 min, 60 min, 90 min

Comparison RCT including Diquafasol After Cataract Surgery

Studies included:: Baek (2016), Park (2017), Inoue (2017), Miyake K. (2017), Cui (2018), Jun (2019), Kim (2021)

- Evaluated time points: All included baseline measurement
    - Baek (2016): 4 weeks, 8 weeks
    - Park (2017): 1 week, 4 weeks, 12 weeks
    - Inoue (2017): 4 weeks, 8 weeks
    - Miyake K. (2017): 4 weeks
    - Cui (2018): 1 week, 4 weeks, 12 weeks
    - Jun (2019): 4 weeks, 12 weeks
    - Kim (2021): 3 weeks, 7 weeks, 15 weeks

	Author	Year	Country	Outcome					Time Points																			Risk of Bias 2					
									Basal	10 min	20 min	30 min	60 min	90 min	220 min	1 wk	2 wk	3 wk	4 wk	5 wk	6 wk	7 wk	8 wk	9 wk	10 wk	12 wk	15 wk	D1	S/D2	D3	D4	D5	overall
	Tsifetaki	2003	Greece	OSDI	TBUT	Schi-I	F.S.3	R.B	x																	x		-	+	+	+	!	-
Cevimelina	Petrone	2002	E.E.U.U	OSDI	TBUT	Schi-I	F.S.3	R.B	x									x			x			x		x		-	+	+	!	!	-
Cevimelina	Ono	2004	Japan	OSDI	TBUT	Schi	F.S.3	R.B	x								x		x									+	+	+	+	+	!
Cevimelina	Leung	2008	China	OSDI	TBUT	Schi	F.S.3	R.B	x								x				x				x			+	+/+	+	+	!	!
No history of cataract	Takamura	2012	Japan	OSDI	TBUT	Schi	F.S.3	R.B	x								x		x									+	+	+	!	+	!
No history of cataract	Matsumoto	2012	Japan	OSDI	TBUT	Schi	F.S.3	R.B	x								x		x		x							+	+	+	+	+	+
No history of cataract	Shimazaki-Den	2013	Japan	OSDI	TBUT	Schi	F.S.3	R.B	x								x		x									!	+	+	+	+	!
No history of cataract	Hwang	2014	South Korea	OSDI	TBUT	Schi	F.S.3	R.B	x										x				x			x		!	+	+	!	+	!
No history of cataract	Gong	2015	China	OSDI	TBUT	Schi	F.S.3	R.B	x								x		x									+	!	!	+	+	!
No history of cataract	Miyake H.	2016	Japan	OSDI	TBUT	Schi	F.S.3	R.B	x	x					x													+	+	+	+	+	+
No history of cataract	Kaido	2018	Japan	OSDI	TBUT	Schi	F.S.3	R.B	x											x								+	+	+	+	+	+
No history of cataract	Fukuoka	2019	Japan	OSDI	TBUT	Schi	F.S.3	R.B	x			x	x	x														+	+	+	+	+	+
After Cataract Surgery	Baek	2016	South Korea	OSDI	TBUT	Schi-I	F.S.3	R.B	x										x				x					!	+	+	+	+	!
After Cataract Surgery	Park	2016	South Korea	OSDI	TBUT	Schi	F.S.3	R.B	x							x			x							x		+	+	!	+	+	!
After Cataract Surgery	Inoue	2017	Japan	OSDI	TBUT	Schi	F.S.3	R.B	x										x				x					+	!	-	+	+	-
After Cataract Surgery	Miyake K.	2017	Japan	OSDI	TBUT	Schi	F.S.3	R.B	x										x									+	!	+	+	+	!
After Cataract Surgery	Cui	2018	South Korea	OSDI	TBUT	Schi	F.S.3	R.B	x							x			x							x		+	+	+	+	+	+
After Cataract Surgery	Jun	2019	South Korea	OSDI	TBUT	Schi	F.S.3	R.B	x										x							x		+	+	+	+	+	+
After Cataract Surgery	Kim	2021	South Korea	OSDI	TBUT	Schi	F.S.3	R.B	x									x				x					x	+	+	+	+	+	+

Figure S1. Included studies representation. Reported outcomes, time points, and risk of bias scores are shown.

Supplementary Materials S2.

Risk of Bias Assessment

The Cochrane Collaboration Risk of Bias 2 (RoB 2) tool was utilized to assess the risk of bias in included studies. This tool evaluated bias related to the randomization process, deviations from intended interventions, missing outcome data, outcome measurement, selection of reported results, and other potential sources of bias[15]. Two review authors (GSR and AKPV) independently classified the risk of bias as "low," "high," or "unclear" (due to insufficient information for assessment) in each domain. A third review author (NKL) resolved any disagreements between review authors **Figure 2S and Figure 3S.**

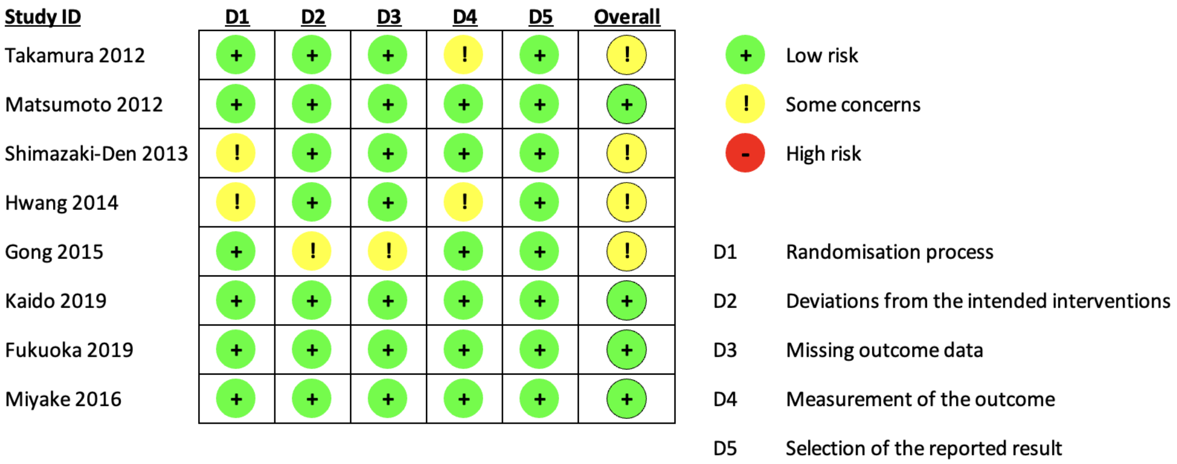

Figure S2. Summary of risk of bias for comparing diquafosol vs. artificial tear s for each included study.

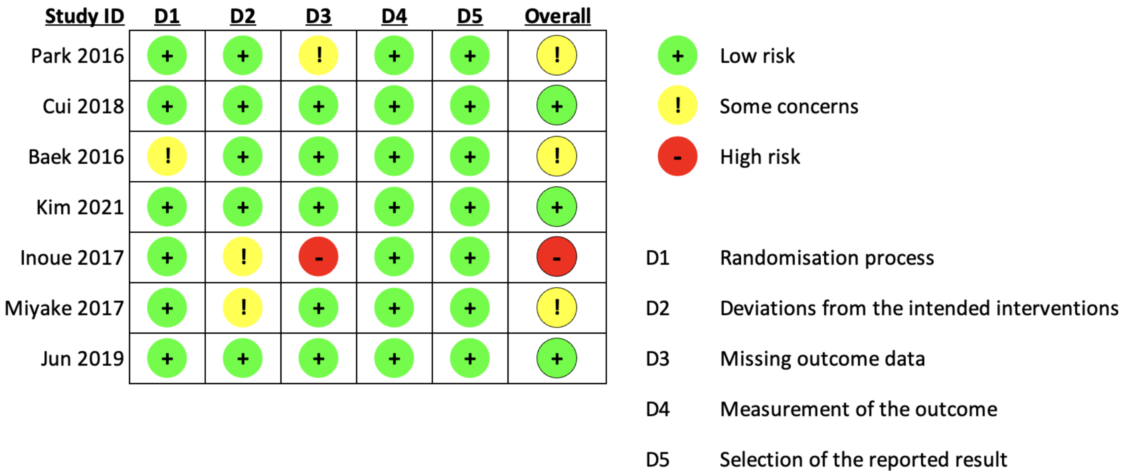

Figure S3. Summary of risk of bias for comparing diquafosol vs. artificial tears after cataract surgery for each included study.

PRISMA

PRISMA 2020 Checklist

Section and Topic	Item #	Checklist item	Location where item is reported
**TITLE**			
Title	1	Identify the report as a systematic review.	Line 1
**ABSTRACT**			
Abstract	2	See the PRISMA 2020 for Abstracts checklist.	Line 11
**INTRODUCTION**			
Rationale	3	Describe the rationale for the review in the context of existing knowledge.	Line 31
Objectives	4	Provide an explicit statement of the objective(s) or question(s) the review addresses.	Line 61
**METHODS**			
Eligibility criteria	5	Specify the inclusion and exclusion criteria for the review and how studies were grouped for the syntheses.	Line 70
Information sources	6	Specify all databases, registers, websites, organisations, reference lists and other sources searched or consulted to identify studies. Specify the date when each source was last searched or consulted.	Line 77
Search strategy	7	Present the full search strategies for all databases, registers and websites, including any filters and limits used.	Supplementary material
Selection process	8	Specify the methods used to decide whether a study met the inclusion criteria of the review, including how many reviewers screened each record and each report retrieved, whether they worked independently, and if applicable, details of automation tools used in the process.	Line 85
Data collection process	9	Specify the methods used to collect data from reports, including how many reviewers collected data from each report, whether they worked independently, any processes for obtaining or confirming data from study investigators, and if applicable, details of automation tools used in the process.	Line 101
Data items	10a	List and define all outcomes for which data were sought. Specify whether all results that were compatible with each outcome domain in each study were sought (e.g. for all measures, time points, analyses), and if not, the methods used to decide which results to collect.	Line 90
	10b	List and define all other variables for which data were sought (e.g. participant and intervention characteristics, funding sources). Describe any assumptions made about any missing or unclear information.	Supplementary material
Study risk of bias assessment	11	Specify the methods used to assess risk of bias in the included studies, including details of the tool(s) used, how many reviewers assessed each study and whether they worked independently, and if applicable, details of automation tools used in the process.	Line 109
Effect measures	12	Specify for each outcome the effect measure(s) (e.g. risk ratio, mean difference) used in the synthesis or presentation of results.	Line 113
Synthesis methods	13a	Describe the processes used to decide which studies were eligible for each synthesis (e.g. tabulating the study intervention characteristics and comparing against the planned groups for each synthesis (item #5)).	Line 101
	13b	Describe any methods required to prepare the data for presentation or synthesis, such as handling of missing summary statistics, or data conversions.	Line 117
	13c	Describe any methods used to tabulate or visually display results of individual studies and syntheses.	Supplementary material
	13d	Describe any methods used to synthesize results and provide a rationale for the choice(s). If meta-analysis was performed, describe the model(s), method(s) to identify the presence and extent of statistical heterogeneity, and software package(s) used.	Line 113
	13e	Describe any methods used to explore possible causes of heterogeneity among study results (e.g. subgroup analysis, meta-regression).	Line 117
	13f	Describe any sensitivity analyses conducted to assess robustness of the synthesized results.	Line 117
Reporting bias assessment	14	Describe any methods used to assess risk of bias due to missing results in a synthesis (arising from reporting biases).	Line 129 & Supplementary material

PRISMA 2020 Checklist

Section and Topic	Item #	Checklist item	Location where item is reported
Certainty assessment	15	Describe any methods used to assess certainty (or confidence) in the body of evidence for an outcome.	Line 20
**RESULTS**			
Study selection	16a	Describe the results of the search and selection process, from the number of records identified in the search to the number of studies included in the review, ideally using a flow diagram.	Line 121
	16b	Cite studies that might appear to meet the inclusion criteria, but which were excluded, and explain why they were excluded.	Line 151, 156
Study characteristics	17	Cite each included study and present its characteristics.	Supplementary material
Risk of bias in studies	18	Present assessments of risk of bias for each included study.	Supplementary material
Results of individual studies	19	For all outcomes, present, for each study: (a) summary statistics for each group (where appropriate) and (b) an effect estimate and its precision (e.g. confidence/credible interval), ideally using structured tables or plots.	Line & Supplementary material
Results of syntheses	20a	For each synthesis, briefly summarise the characteristics and risk of bias among contributing studies.	Supplementary material
	20b	Present results of all statistical syntheses conducted. If meta-analysis was done, present for each the summary estimate and its precision (e.g. confidence/credible interval) and measures of statistical heterogeneity. If comparing groups, describe the direction of the effect.	Line 163
	20c	Present results of all investigations of possible causes of heterogeneity among study results.	Table 1
	20d	Present results of all sensitivity analyses conducted to assess the robustness of the synthesized results.	Table 1
Reporting biases	21	Present assessments of risk of bias due to missing results (arising from reporting biases) for each synthesis assessed.	Supplementary material
Certainty of evidence	22	Present assessments of certainty (or confidence) in the body of evidence for each outcome assessed.	Table 1
**DISCUSSION**			
Discussion	23a	Provide a general interpretation of the results in the context of other evidence.	Line 254
	23b	Discuss any limitations of the evidence included in the review.	Line 307
	23c	Discuss any limitations of the review processes used.	Line 308
	23d	Discuss implications of the results for practice, policy, and future research.	Line 311
**OTHER INFORMATION**			
Registration and protocol	24a	Provide registration information for the review, including register name and registration number, or state that the review was not registered.	Line 66
	24b	Indicate where the review protocol can be accessed, or state that a protocol was not prepared.	Line 69
	24c	Describe and explain any amendments to information provided at registration or in the protocol.	None
Support	25	Describe sources of financial or non-financial support for the review, and the role of the funders or sponsors in the review.	None
Competing interests	26	Declare any competing interests of review authors.	None
Availability of data, code and	27	Report which of the following are publicly available and where they can be found: template data collection forms; data extracted from included studies; data used for all analyses; analytic code; any other materials used in the review.	Supplementary material

PRISMA

PRISMA 2020 Checklist

Section and Topic	Item #	Checklist item	Location where item is reported
other materials			

From: Page MJ, McKenzie JE, Bossuyt PM, Boutron I, Hoffmann TC, Mulrow CD, et al. The PRISMA 2020 statement: an updated guideline for reporting systematic reviews. BMJ 2021;372:n71. doi: 10.1136/bmj.n71.
This work is licensed under CC BY 4.0. To view a copy of this license, visit https://creativecommons.org/licenses/by/4.0/

Supplementary Material S3

Characteristics of Included Studies

The included studies covered three drugs: Pilocarpine, Cevimeline, and Diquafosol.

Clinical trials comparing Pilocarpine versus artificial tears

A randomized controlled trial evaluating the effect of the secretagogue pilocarpine compared to the use of artificial tears was identified by Tsifetaki et al.[40]. This trial took place in Greece and included 85 subjects assigned to three groups through simple randomization. The first group comprised 29 subjects, the second group comprised 28 subjects, and all subjects in these two groups completed the follow-up. A third group underwent punctal occlusion, with 28 subjects assigned, of which 26 completed the follow-up. Since only one clinical trial was identified, it was not possible to make a quantitative synthesis. The follow-up period was 12 weeks with two measurements: baseline and one at the end of the follow-up. The outcome measured was rose bengal staining and fluorescein stain score.

Clinical trials with Cevimeline

Three clinical trials were identified that included Cevimeline as an intervention. The first study, conducted by Petrone et al.[41], aimed to assess the use of Cevimeline versus placebo for treating dry eye and dry mouth in subjects with SS. This clinical trial included 197 subjects randomized into three groups: the placebo group with 70 subjects and two intervention groups. The first intervention group comprised 65 subjects receiving 15 mg of Cevimeline three times daily, while the second intervention group had 62 subjects receiving 30 mg of Cevimeline three times daily. The follow-up period for this clinical trial was 3, 6, 8, and 12 weeks, with the reported outcome being the STT. Only patients receiving 30 mg of Cevimeline were included in this study, as it represents the standard dose and is consistent with the other identified clinical trials.

Ono et al.[18] conducted the second clinical trial. This trial, also targeting dry eye and dry mouth in SS subjects, employed a randomized, double-blind design. The trial included 60 subjects randomized into three groups: the placebo group with 20 subjects and two intervention groups. The first intervention group had 20 subjects receiving 20 mg of Cevimeline three times daily, while the second group comprised 20 subjects receiving 30 mg of Cevimeline three times daily. No information regarding subject loss during the study was reported. The follow-up period for this clinical trial was 2 and 4 weeks, with reported outcomes including TBUT, STT (specific type not specified), and Rose Bengal and Fluorescein stain score.

Leung et al.[42] conducted the third study identified. This randomized controlled crossover trial included a 4-week washout period. The trial aimed to evaluate Cevimeline versus placebo for treating dry eye and dry mouth in SS subjects, conducted at the Hong Kong dental school, focusing primarily on Xerostomia outcomes. The specific dry eye outcome, assessed using the Chinese Dry Eye Questionnaire, did not provide extractable information.

Of these three clinical trials, it was not possible to conduct a quantitative synthesis because the same outcomes or time points were not shared; however, all three studies report better performance by Cevimelinae

Clinical trials comparing Diquafosol versus artificial tears.

Eight clinical trials involved comparing Diquafosol against artificial tears as an intervention. The first study identified was conducted by Takamura et al.[34] with a randomized controlled trial design with double masking. A total of 286 subjects were included and distributed into two groups; the diquafosol intervention group consisted of 143 subjects, while the artificial tears group was assigned 143 subjects. Of the total subjects included, 99% completed the study, with two dropouts in the diquafosol group due to adverse effects and four in the artificial tears group due to therapeutic failure and two due to the adverse impacts). The study lasted for four weeks, with measurements taken at 2 and 4 weeks, and the outcomes measured were Rose Bengal staining and fluorescein stain score.

The second study identified was conducted by Matsumoto et al.[30]with a randomized controlled trial design with double masking. 192 subjects were divided into two groups ;
the diquafosol intervention group consisted of 96 subjects, while the artificial tears group was assigned 94 subjects. There were no reported losses among the total subjects included in the study. The study lasted six weeks, with measurements taken at baseline, week 2, week 4, and week 6. The outcomes measured were tear film breakup time, Rose Bengal staining, and fluorescein stain score.

Shimazaki-Den et al.[31], an open-label randomized controlled trial design, was the third study. A total of 17 subjects were included and distributed into 2 groups: the diquafosol intervention group consisted of 9 subjects, while the artificial tears group was assigned 8 subjects. There were no reported losses among the total number of subjects included in the study. The study lasted for 4 weeks, with measurements taken at baseline, week 2, and week 4. The outcomes measured were TBUT and fluorescein stain score.

Hwang et al.[33] conducted the fourth study identified. It had a randomized, controlled open-label trial design. 150 subjects were included and divided into three groups: 50 subjects in the Diquafosol intervention group and 50 in the artificial tears group. Of th e

subjects included in the study, 87% completed the protocol. Diquafosol group had five subjects who did not complete the follow-up, while the artificial tears group had eight subjects who did not complete the follow-up, all due to therapeutic failure. The study lasted 12 weeks, with measurements taken at baseline, week 4, week 8, and week 12. The outcomes measured were the OSDI, TBUT, STT-1, Rose Bengal Staining, and fluorescein staining scores.

The fifth study identified was conducted by Gong et al.[32]. It had a randomized, controlled open-label trial design. A total of 497 subjects were divided into two groups: 246 subjects in the Diquafosol intervention group and 251 subjects in the artificial tears group. Of the total subjects included in the study, 98% completed the follow-up. In the Diquafosol group, 11 subjects were discontinued due to adverse effects, while in the artificial tears group, 12 subjects did not complete the follow-up. The study lasted 4 weeks, with measurements taken at baseline, week 2, and week 4. The outcomes measured were TBUT, Rose Bengal Staining, and fluorescein stain score.

Miyake et al.[37] conducted the sixth study identified. It had a randomized, controlled double-masked trial design. A total of 50 subjects were included and divided into two groups: 25 subjects in the Diquafosol intervention group and 25 in the artificial tears group. All subjects included in the study completed the follow-up. The study lasted for 220 minutes, with measurements taken at baseline, 10, and 220 minutes. The outcome measured was STT-1.

Kaido et al.[43] conducted the seventh study identified. It had a randomized, controlled open-label trial design. A total of 27 subjects were included and divided into two groups: 12 subjects in the Diquafosol intervention group and 15 subjects in the artificial tears group. All subjects included in the study completed the follow-up. The study lasted 5 weeks, with measurements taken at baseline and five weeks. The outcomes measured were OSDI and TBUT.

Fukuoka et al.[44] conducted the eighth study identified. It had a randomized, controlled open-label trial design. A total of 94 subjects were divided into two groups: 47 subjects in the Diquafosol intervention group and 47 subjects in the artificial tears group. All subjects included in the study completed the follow-up. The study lasted 90 minutes, with measurements taken at baseline, 30 minutes, 60 minutes, and 90 minutes. The outcome measured was TBUT.

Diquafosol versus artificial tears following cataract surgery.

The first study identified was conducted by Baek et al.[39]. It was conducted in South Korea and had an open-label randomized controlled trial design. The study included a total of 34 post-cataract surgery participants divided into two groups: the Diquafosol intervention group consisted of 17 subjects. The artificial tears group had 17 subjects

assigned to it, with no reported losses to follow-up. The study lasted eight weeks, with measurements taken at baseline, week 4, and week 8. The outcomes measured were TBUT, STT-1, and fluorescein stain scores.

The second study identified was conducted by Park et al.[16]. It was conducted in South Korea with an open-label randomized controlled trial design among post-cataract surgery subjects. A total of 63 subjects were included and divided into two groups: the Diquafosol intervention group consisted of 30 subjects. The artificial tears group had 33 subjects assigned to it, with no reported losses to follow-up. The study lasted 12 weeks, with measurements taken at baseline, week 1, week 4, and week 12. The outcomes measured were the OSDI, TBUT, STT-1, and fluorescein stain scores.

The third study identified was conducted by Inoue et al.[36]. It was conducted in Japan with an open-label randomized controlled trial design among post-cataract surgery subjects. A total of 42 subjects were included and divided into two groups: the Diquafosol intervention group, which consisted of 20 subjects. The artificial tears group had 22 subjects assigned to it, with no reported losses to follow-up. The study lasted eight weeks, and measurements were taken at baseline, week 4, and week 8. The outcomes measured were TBUT, STT-1, and fluorescein stain scores.

The fourth study identified was conducted by Miyake et al.[37]. It was conducted in Japan with an open-label randomized controlled trial design among post-cataract surgery subjects. A total of 154 subjects were included, divided into two groups: the Diquafosol intervention group consisted of 75 subjects. In comparison, the artificial tears group had 79 subjects assigned to it, with no reported losses to follow-up. The study lasted for four weeks, with measurements taken at baseline and week 4. The outcomes measured were the OSDI, TBUT, and fluorescein stain scores.

Cui et al.[35] conducted the fifth study identified with an open-label randomized controlled trial design among post-cataract surgery subjects. Ninety-four subjects were included and divided into two groups: the Diquafosol intervention group consisted of 50 subjects. The artificial tears group had 44 subjects assigned to it, with no reported losses to follow-up. The study lasted 12 weeks, with measurements taken at baseline, week 1, week 4, and week 12. The outcomes measured were OSDI, TBUT, and STT-1.

The sixth study identified was conducted by Jun et al.[38]. It was conducted in an open-label randomized controlled trial design among post-cataract surgery subjects. A total of 117 subjects were included, divided into three groups: the Diquafosol intervention group consisted of 41 subjects. In comparison, the artificial tears group had 38 subjects assigned to it, with no reported losses to follow-up. The study lasted 12 weeks, and measurements were taken at baseline, week 4, and week 12. The outcomes measured were OSDI, TBUT, STT-1, and fluorescein stain scores.

The seventh study identified was conducted by Kim et al.[46]. It was conducted in South Korea with a double-masked randomized controlled trial design among post-cataract surgery subjects. A total of 56 subjects were included and divided into two groups: the Diquafosol intervention group consisted of 28 subjects. The artificial tears group had 28 subjects assigned to it, with no reported losses to follow-up. The study lasted 15 weeks, with measurements taken at baseline, week 3, week 8, and week 15. The outcomes measured were OSDI, TBUT, and STT-1.

Other Effects of Interventions

Diquafosol 3% Vs. Artificial Tears

Rose Bengal Staining

Three clinical trials evaluated the effect of Diquafosol 3% versus Artificial Tears after two weeks of treatment [21,22,24]. The combined data from all trials resulted in 477 subjects assigned to Diquafosol 3% and 480 subjects assigned to Artificial Tears treatment, yielding a MD, -0.21, 95% CI, -0.43 to 0.01, **Figure 4S**. Four trials assessed the effect of Diquafosol 3% versus Artificial Tears after four weeks of treatment [21–24]. The combined data from all trials resulted in 538 subjects assigned to Diquafosol 3% and 523 subjects assigned to Artificial Tears treatment, yielding a MD, -0.54, 95% CI, -0.87 to -0.21, **Figure 5S**. In the two-week comparison, the p-value was 0.06, indicating no statistically significant difference between the use of Diquafosol 3% and Artificial Tears at this time. However, in the four-week comparison, the Z-value was 3.20, with a p-value of 0.01, resulting in a MD, -0.54, 95% CI -0.87 to -0.21, showing a better response in the Diquafosol 3% group.

	Diquafosol 3%			Artificial Tear				Mean Difference
Study or Subgroup	Mean	SD	Total	Mean	SD	Total	Weight	IV, Random, 95% CI
Gong 2015	3.12	1.7	237	3.28	1.52	244	58.0%	−0.16 [−0.45, 0.13]
Matsumoto 2012	3.46	1.74	96	3.5	1.83	94	18.7%	−0.04 [−0.55, 0.47]
Takamura 2012	3.92	2.28	144	4.41	1.59	142	23.3%	−0.49 [−0.95, −0.03]
**Total (95% CI)**			**477**			**480**	**100.0%**	**−0.21 [−0.43, 0.01]**

Heterogeneity: $Tau^2 = 0.00$; $Chi^2 = 2.00$, df = 2 (P = 0.37); $I^2 = 0\%$
Test for overall effect: Z = 1.91 (P = 0.06)

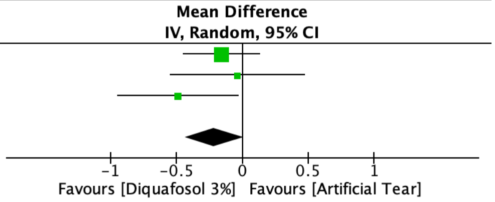

Figure S4. Forest plot of comparison: Diquafosol 3% versus artificial tears outcome: Rose Bengal after 2 weeks of treatment

	Diquafosol 3%			Artificial Tear				Mean Difference	
Study or Subgroup	Mean	SD	Total	Mean	SD	Total	Weight	IV, Random, 95% CI	Year
Matsumoto 2012	2.8	1.95	96	3.54	2.03	94	19.3%	−0.74 [−1.31, −0.17]	2012
Takamura 2012	3.04	2.28	144	4.02	2.14	142	21.5%	−0.98 [−1.49, −0.47]	2012
Hwang 2014	1.83	1.06	50	2.26	0.7	50	29.5%	−0.43 [−0.78, −0.08]	2014
Gong 2015	2.5	2	248	2.7	1.9	237	29.8%	−0.20 [−0.55, 0.15]	2015
**Total (95% CI)**			**538**			**523**	**100.0%**	**−0.54 [−0.87, −0.21]**	

Heterogeneity: $Tau^2 = 0.06$; $Chi^2 = 7.05$, df = 3 (P = 0.07); $I^2 = 57\%$
Test for overall effect: Z = 3.20 (P = 0.001)

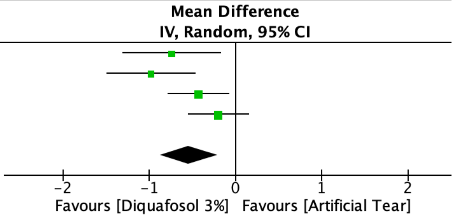

Figure S5. Forest plot of comparison: Diquafosol 3% versus artificial tears outcome: Rose bengal after 4 weeks of treatment

Diquafosol 3% Vs Artificial tears after cataract surgery.

STT

A MD analysis with a random-effects model was used for this outcome. Two clinical trials evaluated the effect of Diquafosol 3% versus Artificial Tears in subjects post-cataract surgery after one week of treatment, Park and Cui (79,82). The combined data from these trials resulted in 80 subjects assigned to Diquafosol 3% and 77 subjects assigned to Artificial Tears treatment, yielding a MD, 0.26, 95% CI,0.11 to 0.42, **Figure 6S**. It was also possible to compare this outcome at 4 weeks of treatment since four clinical trials reported the outcome [29,30,32,33]. The combined data from these trials resulted in 141 subjects assigned to Diquafosol 3% and 137 subjects assigned to Artificial Tears treatment, yielding a MD of 0.630, 95% CI, 0.37 to 0.89, **Figure 7S**.

Furthermore, the outcome was evaluated at 12 weeks in three studies[29,32,33]. The combined data from these trials resulted in 121 subjects assigned to Diquafosol 3% and 115 subjects assigned to Artificial Tears treatment, yielding a MD, 1.09, 95% CI, 0.09; 2.10, **Figure 19**. The Z= 3.31, p-value of 0.0009 in the one week comparison, 4.70, p-value of 0.00001 in the 4-week comparison, and Z=2.13 with a p-value of 0.03 in the 12-week comparison, respectively, showing a better response in the Diquafosol 3% group.

	Diquafosol 3%			Artificial Tear				Mean Difference
Study or Subgroup	Mean	SD	Total	Mean	SD	Total	Weight	IV, Random, 95% CI
Cui 2018	4.6	4.06	50	4.82	3.46	44	1.1%	−0.22 [−1.74, 1.30]
Park 2016	2.03	0.37	30	1.76	0.25	33	98.9%	0.27 [0.11, 0.43]
**Total (95% CI)**			**80**			**77**	**100.0%**	**0.26 [0.11, 0.42]**

Heterogeneity: $Tau^2 = 0.00$; $Chi^2 = 0.39$, df = 1 (P = 0.53); $I^2 = 0\%$
Test for overall effect: Z = 3.31 (P = 0.0009)

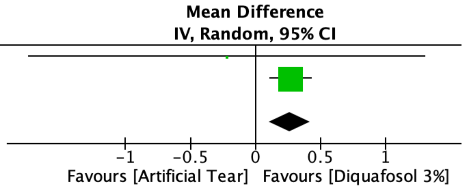

Figure S6. **Forest Plot Comparing Diquafosol 3% vs. Artificial Tears in Postoperative Cataract Patients Outcome: Schirmer Test After Four Weeks of Treatment.**

	Diquafosol 3%			Artificial Tear				Mean Difference
Study or Subgroup	Mean	SD	Total	Mean	SD	Total	Weight	IV, Random, 95% CI
Cui 2018	6.28	5.06	50	6.01	4.06	44	2.0%	0.27 [−1.58, 2.12]
Inoue 2017	11.71	7.26	20	10.21	6.24	22	0.4%	1.50 [−2.61, 5.61]
Jun 2019	11.4	8	41	10.6	6.1	38	0.7%	0.80 [−2.32, 3.92]
Park 2016	3.22	0.62	30	2.59	0.43	33	96.9%	0.63 [0.36, 0.90]
**Total (95% CI)**			**141**			**137**	**100.0%**	**0.63 [0.37, 0.89]**

Heterogeneity: $Tau^2 = 0.00$; $Chi^2 = 0.33$, df = 3 (P = 0.95); $I^2 = 0\%$
Test for overall effect: Z = 4.70 (P < 0.00001)

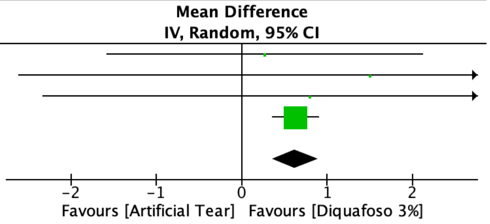

Figure S7. **Forest Plot Comparing Diquafosol 3% vs. Artificial Tears in Postoperative Cataract Patients Outcome: Schirmer Test After Twelve Weeks of Treatment.**
